# Defining Cell Cluster Size by Dielectrophoretic Capture at an Array of Wireless Electrodes of Several Distinct Lengths

**DOI:** 10.3390/mi10040271

**Published:** 2019-04-23

**Authors:** Joseph T. Banovetz, Min Li, Darshna Pagariya, Sungu Kim, Baskar Ganapathysubramanian, Robbyn K. Anand

**Affiliations:** 1 Department of Chemistry, Iowa State University, 1605 Gilman Hall, 2415 Osborn Drive, Ames, IA 50011, USA; joebano@iastate.edu (J.T.B.); minl@iastate.edu (M.L.); darshna@iastate.edu (D.P.); skim@iastate.edu (S.K.); 2 Department of Mechanical Engineering, Iowa State University, 2043 Black Engineering, 2529 Union Drive, Ames, IA 50011, USA; baskarg@iastate.edu

**Keywords:** cell patterning, cell cluster, dielectrophoresis, wireless electrode array, bipolar electrode

## Abstract

Clusters of biological cells play an important role in normal and disease states, such as in the release of insulin from pancreatic islets and in the enhanced spread of cancer by clusters of circulating tumor cells. We report a method to pattern cells into clusters having sizes correlated to the dimensions of each electrode in an array of wireless bipolar electrodes (BPEs). The cells are captured by dielectrophoresis (DEP), which confers selectivity, and patterns cells without the need for physical barriers or adhesive interactions that can alter cell function. Our findings demonstrate that this approach readily achieves fine control of cell cluster size over a broader range set by other experimental parameters. These parameters include the magnitude of the voltage applied externally to drive capture at the BPE array, the rate of fluid flow, and the time allowed for DEP-based cell capture. Therefore, the reported method is anticipated to allow the influence of cluster size on cell function to be more fully investigated.

## 1. Introduction

**Scheme 1:**
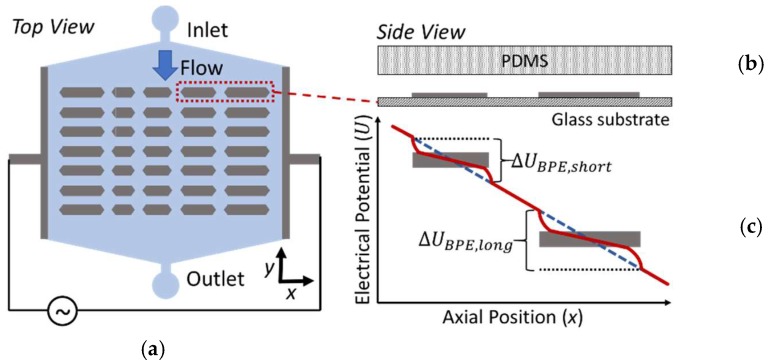
(**a**) Top view schematic of the microfluidic chamber, array of bipolar electrodes (BPEs), and driving electrodes (not to scale). (**b**) Side view schematic of a segment of the microfluidic chamber (along the *x* direction) containing two wireless BPEs of different lengths. (**c**) Illustration of the electrical potential profile of the solution and of the two BPEs depicted in (**b**), showing the dependence of $\Delta U_{BPE}$ on BPE length.

In this paper, we demonstrate a method for patterning biological cells that achieves control over the size of small cell clusters (2–12 cells) by their dielectrophoretic capture at an array of wireless bipolar electrodes (BPEs) having several distinct lengths (Scheme 1a,b). Specifically, we show that capture force, and therefore cluster size, is positively correlated with BPE length. This relationship arises from an increase in the electrical potential difference across the solution in contact with each BPE ($\Delta U_{BPE}$) on its length (Scheme 1c). This finding is significant because control over cluster size is achieved without physical barriers or surfaces with pre-patterned biomolecules, and therefore, the cluster size is readily tunable via voltage and fluid flow rate. Further, dielectrophoresis (DEP) itself provides selectivity based on the frequency-dependent dielectric response of cells arising from their morphology and composition. Finally, relative to DEP approaches that employ interelectrode spacing to control capture force, BPE length gives finer control. Therefore, DEP at BPE arrays provides a simple experimental handle to define both the number and type of cells patterned. Here, we describe the dependence of the number of cells captured in each cluster on BPE length, the magnitude and duration of the voltage applied externally to drive DEP-based capture at the array, and the flow rate of the cell solution. 

The ability of cells to respond to physical and chemical cues leads to a dependence of their behavior on the cell microenvironment. Therefore, the study of normal and disease states, responses to toxins or therapeutics, and the role of cell microenvironment itself, necessitate an ability to develop systems that recapitulate the structure of native biological tissues. For example, the size of cell clusters is of major importance to widely separated areas of clinical interest, including cancer biology and pancreatic function. Cancer cells undergo morphological changes conducive to metastasis following diminishing cell-cell adhesion [1,2]. However, once present in the bloodstream, cancer cells exhibit greater metastatic potential when present as clusters rather than as single cells due to their ability to avoid apoptosis [2,3]. Furthermore, it has been suggested that cancer cell clusters are more likely to experience physical cues triggering tumor growth than single cells [4]. Such circulating tumor cell clusters ranging in size from 2 to 45 cells have been isolated from patients and are an indicator of poor prognosis [5]. Therefore, clusters of cancer cells in this size range are an important focus of therapeutic and diagnostic research. Cell clustering is likewise important in pancreatic function. For example, cultured pancreatic precursor cells release more insulin when clustered than singly [6], and clusters 100–120 µm in diameter produce insulin at peak efficiency [7].

Patterning of cells has been accomplished using a variety of physical and chemical methods. Cluster size has been defined physically by depositing cells into an array of microwells in combination with either hydraulic forces [8] or specific adhesive interactions [9] to retain them. Microwells are simple to implement and allow high resolution patterning. However, microwells have three important limitations. First, they do not select for cell type on their own. While specific cells can be targeted through adhesive interactions, the selectivity of such interactions is not readily tunable. Second, the size of the trapped clusters is necessarily defined by the diameter of the microwells; while a range of cluster sizes could potentially be accomplished by using a range of well diameters, further modification of cluster size in situ is prohibited by fixed microwell dimensions. Finally, the most important limitation is that the physical constraints imposed by the wells have the potential to affect cell behavior [10]. Another physical method, microcontact printing, utilizes microdroplets to contain cells, and “print” them onto a substrate [11]. Like microwells, this approach does not include any inherent selectivity and limits in situ control of cell cluster size because the number of cells in the droplet is determined at the time of deposition. Finally, acoustophoresis, which uses standing acoustic waves generated by piezoelectric materials, confers size selectivity and fine control over location [12,13,14]. Acoustophoresis has some advantages relative to other physical methods in that it does not rely on geometric constraints and that it provides tunable selectivity based on cell size and compressibility. Acoustophoresis requires complex fabrication of a piezoelectric substrate, however, and selectivity is limited when attempting to separate cells with similar mechanical properties.

Cell patterning can also be achieved chemically by pre-patterning cell adhesion promoters or inhibitors. For example, mammalian cells will preferentially adhere to proteins such as fibronectin, laminin and collagen coated on glass or polymer substrates [15]. Patterning these proteins on a substrate will cause the cells to congregate in the areas coated. Such adhesive interactions are also used as a method to retain cells that are initially patterned by other methods [9,16]. While chemically patterned substrates have been used to examine many cell behaviors [17,18], their limitations include that the cell cluster size is pre-determined by the geometry of the patterned spots, thereby precluding in situ control, and that certain adhesive interactions may impact cell behavior. Finally, the degree of selectivity depends on the nature of the binding interaction, which can be non-specific (e.g., fibronectin) or highly specific (e.g., antibody). 

Magnetic patterning makes use of specific chemical interactions to label cells with magnetic particles for manipulation [19]. Magnetic patterning allows for coarse control over cell location without the need for physical barriers, and in situ manipulation to adjust cell location is possible. Selectivity is conferred by directing the magnetic tags to a cell surface antigen and is therefore, highly specific and fixed (not tunable during patterning). 

Cell manipulation techniques that employ patterned light, such as optical tweezers and optoelectronics provide fine control over cell location [20]. However, optical tweezers are frequently utilized to manipulate one cell at a time because resolution suffers when operated on a larger scale [8]. Moreover, in this method, a laser exposes the cells to high power levels, which can negatively affect cell function, unless a patterned (e.g., ring shaped) beam is employed [21,22]. Optoelectronic tweezers (OET), also known as optically induced DEP (ODEP), controls cell position with lower power exposure to the cells than optical tweezers [23]. These methods avoid physical barriers and labels that disturb cell function, allow cell location to be adjusted after initial patterning, and in the case of OET, can be made selective. However, the need for advanced optical components presents a barrier to broad application in cell research.

Similar to these methods, DEP does not rely on protein affinity or physical constraints, but instead, exerts electrostatic force on biological cells based on an interaction between an electric field gradient and a field-induced dipole in the cell. Cellular DEP has been described in detail elsewhere [24,25] and is briefly summarized here. The time averaged dielectrophoretic force, 〈$F_{DEP}$〉, exerted on a spherical polarizable particle by an electric field is given by the following equations.

(1) $$\langle F_{DEP}\rangle =2\pi r^{3}\varepsilon_{m}Re\left[K\left(\omega \right)\right]\nabla {\left|E\right|}^{2}$$

(2) $$K\left(\omega \right)=\left(\varepsilon_{p}^{*}-\varepsilon_{m}^{*}\right)/\left(\varepsilon_{p}^{*}+2\varepsilon_{m}^{*}\right)$$

where r is the particle radius, $\varepsilon_{m}$ is the permittivity of the medium, E and ω are the magnitude and frequency of the electric field, respectively. Re[K(ω)] is the real part of the Clausius-Mossotti factor, which is a frequency-dependent term that compares the complex permittivities of the particle ($\varepsilon_{p}^{*}$) and medium ($\varepsilon_{m}^{*}$). The sign of this term determines whether the particle is attracted (Re[K(ω)]>0, positive, pDEP) or repelled (Re[K(ω)]<0, negative, nDEP) from regions of higher electric field strength, such as an electrode tip.

Unlike a uniform spherical particle, the sign and magnitude of Re[K(ω)] for a biological cell best fits a core-shell model that represents the cytoplasm and cell membrane, and therefore, depends sensitively on cell size, morphology, and composition. In this way, DEP exhibits cell type selectivity that is tunable via electric field frequency [26,27]. Despite exposure to electric field gradients, the effect of DEP on cell viability is minimal [24]. DEP has been used to pattern a wide variety of cells [24,28,29,30] and to form well-defined cell clusters [31,32]. Previously, we reported DEP capture of breast cancer cells on a wireless BPE array for separation of tumor cells from blood cells [33] and for analysis of cell contents [34]. In the former case, we demonstrated a BPE array of uniform lengths in which MDA-MB-231 cells were separated from Jurkat model white blood cells based on their DEP response. In both cases, the BPE tips defined tens to thousands of capture points without necessitating wire leads to each. Especially relevant to cell patterning is that cancer cells were captured singly by using pockets embedded in microchannel walls overlying the BPE array as a geometric restriction [33,35]. A key point is that consistently-sized clusters resulted even in the absence of these physical constraints. This result is in agreement with previous reports demonstrating that a large array of BPEs can be uniformly polarized under both Direct Current (DC) [36,37] and Alternating Current (AC) [38,39] conditions. Here, cluster size is defined by a balance between DEP capture force (*F*_DEP_), which decays with distance from each BPE tip, and the drag force (*F*_drag_) imposed by fluid flow. In this paper, we demonstrate that the size of clusters of an invasive breast cancer cell line (MDA-MB-231) captured by DEP at a BPE array can be controlled through BPE length along electric field lines. Under conditions in which a cell-containing solution is flowed across a BPE array, we examine the relative influence of BPE length, the magnitude and duration of an externally applied voltage, and the fluid flow rate on the resultant number of cells captured to form clusters at each BPE tip. We find that under a single applied voltage and flow rate, the average number of cells per cluster is positively correlated with BPE length and that the rate of accumulation slows over a period of 5 min. Under the conditions investigated, cluster size was more sensitive to voltage and flow rate than to BPE length. This approach is advantageous in that (i) voltage and flow rate define a coarse range in the number of cells captured, while BPE length provides fine control, (ii) device fabrication is simple and robust, (iii) no physical barriers are needed, and since it employs DEP, (iv) cell viability is preserved, and (v) the selectivity for cell type is tunable via electric field frequency.

## 2. Materials and Methods

### 2.1. Chemicals

The silicone elastomer and curing agent (Sylgard 184), bovine serum albumin (BSA) (biotech grade), and 0.25% Trypsin EDTA (1X) were purchased from Fisher Scientific (Thermo Fisher Scientific, Inc., Waltham, MA, USA). The DMEM/F12 cell culture medium, dextrose (D-glucose), sucrose, Pluronic F-108 and 1.0 M Tris·HCl stock were obtained from Sigma-Aldrich, Inc. (St. Louis, MO, USA). All dilutions were conducted with Type 1 water (18.2 MΩ·cm). DEP buffer was comprised of 8.0% sucrose, 0.3% dextrose, and 0.1% BSA in 1.0 mM Tris buffer (pH 8.1, conductivity 72 µS/cm).

### 2.2. Cell Culture

MDA-MB-231 cells were obtained from ATCC (American Type Culture Collection, Manassas, VA, USA). They were cultured in DMEM/F12 with 10% fetal bovine serum supplementation at 37 °C and 5% CO_2_. All cells were subcultured every 2−3 days to maintain the concentration of cells at less than 80% confluence. In preparation for DEP experiments, MDA-MB-231 cells were detached from culture flask using 0.25% Trypsin-EDTA (1X), followed by pelleting by centrifugation (1100 rpm, 5 min) and resuspension in 5.0 mL DEP buffer. Pelleting and resuspension was repeated to wash cells twice in DEP buffer before DEP capture experiments. The final cell concentration for each experiment was 2 × 10^6^ cells/mL.

### 2.3. Device Fabrication

The hybrid PDMS/glass microfluidic device with embedded thin metal film electrodes was fabricated by soft lithography [40]. Briefly, a glass microscope slide (75 mm × 25 mm × 1 mm) with a thermally evaporated 10 nm-thick Cr adhesion layer and 100 nm-thick Au film was patterned using a positive photoresist (AZP4620, MicroChemicals GmbH, Ulm, Germany), wet etched with 4% KI/1% I_2_ followed by chrome etchant (Sigma Aldrich, St. Louis, MO, USA), and then rinsed with DI water. The photoresist was then stripped with acetone and the slide dried with a stream of N_2_. Separately, the PDMS monolith was patterned by pouring PDMS precursor (10:1 base:curing agent) over a photolithographically patterned film of 50 μm-thick SU-8 2050 on a Si wafer. The PDMS was cured for 24 h at room temperature, the device cut from the mold, and inlet and outlet reservoirs made with a biopsy punch. The patterned slide and PDMS monolith were exposed to an air plasma (medium power, PDC-001, Harrick Plasma, Ithaca, NY, USA) for 1 min. A drop of ethanol was placed between the two pieces to allow adjustment of position during alignment. The device was then dried in a 70 °C oven for 1 h to drive off excess ethanol and to promote sealing. Finally, the device was filled with 3.0 μM Pluronic in DEP buffer and incubated at room temperature overnight. The Pluronic coating decreased non-specific adhesion of the cells to the surfaces of the device. Each device was rinsed with DEP buffer by replacing the solution in the inlet reservoir and withdrawing at 1.0 μL/min for 5 min prior to cell capture experiments.

### 2.4. Device Dimensions

The device dimensions were as follows. The microchamber was 3.6 mm wide, 2.6 mm long, and 50 μm tall. 40 μm-diameter pillars supporting the chamber ‘ceiling’ were aligned over the central segment of each BPE. The device inlet reservoir was 5.0 mm in diameter to facilitate exchange of inlet solutions and the outlet was a 1.0 mm reservoir connected to a syringe pump with tubing. The electrodes were designed with nominal lengths of 100, 160, 275, 400, and 500 μm and had rounded triangular tips. The spacing between electrodes along both *x*- and *y*- axes was 160 μm. Each row (*x*-direction) was comprised of one BPE of each length, and each column (*y*-direction), 20 identical BPEs. Note that Scheme 1a is not to scale and that only a subset of BPEs is depicted. A pair of coplanar driving electrodes were exposed to the interior of the chamber at the sides, flanking the array.

### 2.5. DEP Capture

The device was designed to allow cells suspended in DEP buffer to flow through and be attracted to the BPE tips in pDEP mode. AC voltage to drive the DEP response was applied to the driving electrodes using a Tektronix AFG3011C waveform generator (Tektronic, Beaverton, OR, USA) and Trek model 2205 amplifier (Trek, Lockport, NY, USA). The AC frequency was maintained at 70 kHz, as determined by our previous work [32], to ensure the MDA-MB-231 cells experienced strong pDEP. The devices were imaged using a Nikon AZ100 microscope. Flow was induced using a Pico Plus Elite syringe pump (Harvard Apparatus, Holliston, MA, USA) paired with a 500 μL glass syringe (Hamilton Company, Reno, NV, USA) in withdrawal mode. Micrographs of the array were collected during DEP capture and the cells were counted manually. 

## 3. Results and Discussion

### 3.1. Theoretical Background

**Figure 1:**
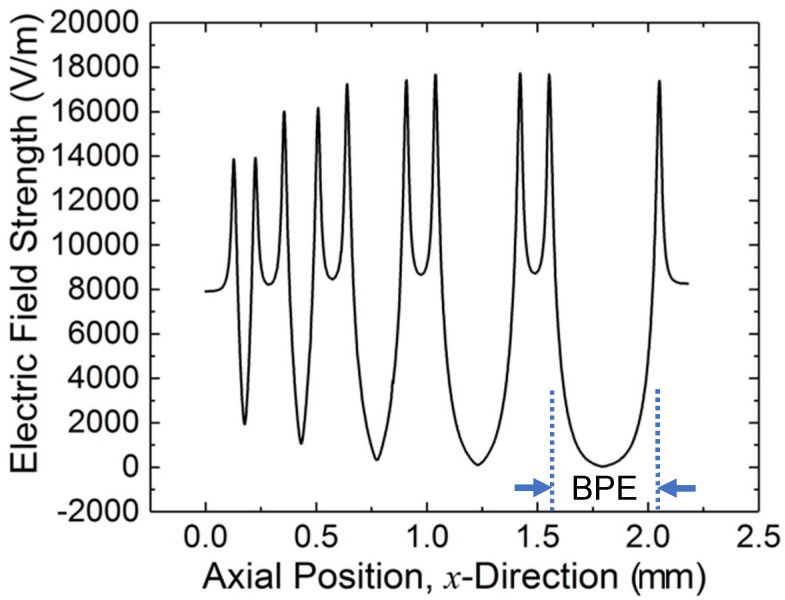
Simulated electric field norm along a 2D cut line taken along the *x*-direction at 9 μm above the chamber floor across 100, 160, 275, 400, and 500 μm-long BPEs. The position of the 500 μm-long BPE is labeled with dashed lines for clarity.

Austin and coworkers previously described pDEP of DNA at an array of floating conductors of uniform size and shape—a technique which they called ‘electrodeless DEP’ [41]. Further, Hamdi et al. reported nDEP capture and fusion (by electric pulse) of pairs of cells at a uniform array of non-connected gold pads [42]. In the current study, cells were captured using DEP on a wireless BPE array that incorporated a range of electrode lengths (top and side views, Scheme 1a,b). Scheme 1c depicts the electrical potential profile of the solution in the absence (dashed line) and presence (solid line) of BPEs under an externally applied voltage bias. Two BPEs are depicted, and represent conductive strips of two distinct lengths defined from a thin metal film. Each BPE floats to a potential ($U_{BPE}$) intermediate to that in the solution contacting it, thus resulting in an interfacial potential difference at each of its opposing ends (BPE tips). The total potential difference in the solution phase along the BPE, $\Delta U_{BPE}$, can be described by the following equation.

(3) $$\Delta U_{BPE}=\frac{l_{BPE}\Delta U_{tot}}{l_{chamber}}$$

here, $\Delta U_{tot}$ is the total potential difference applied between the external driving electrodes. $l_{BPE}$ and $l_{chamber}$ are the length of the BPE and the chamber (*x*-direction, Scheme 1a), respectively. In such an electric field, polarization of the BPE and charging of the electrical double layer at its surface depolarizes the solution it contacts and generates electric field maxima at its poles (Figure 1). A longer BPE induces greater field maxima thereby exerting greater *F*_DEP_, which in turn allows it to capture more cells than a shorter electrode. By varying the length of the BPEs within the wireless array, a range of magnitudes in electric field maxima can be accomplished in the same device. Thus, a range of cell cluster sizes can be achieved. In the following subsections, we examine the influence of $\Delta U_{tot}$, volumetric flow rate, and BPE length on cluster size. 

Figure 1 is a plot of the total electric field strength across a cross section (*xz* plane) of the microfluidic chamber at 9 μm above 100, 160, 275, 400, and 500 μm-long BPEs in a 3D numerical simulation (Multiphysics, COMSOL, Inc., Burlington, MA, USA). The dimensions of the chamber segment were 2180 μm wide, 750 μm long and 50 μm tall. The floor (bottom), ceiling (upper), and side boundaries had zero surface charge. The inlet and outlet boundaries were open to flux of ions. Each BPE was represented as a boundary with a floating potential, such that the integral of electric flux over the BPE surface was equal to zero (the net charge on the BPE). This geometry was discretized into a mesh with size ranging from 0.44 μm (near the electrode tips) to 43.6 μm (near the chamber walls). The dielectric constant of the medium was 80 (water). The sides of the chamber had a voltage bias between them of 9.9 V, which is equivalent to 16 V (32 V_pp_) over the full 3.6 mm wide array. Under these conditions, the AC frequency is too high to allow significant accumulation of charge in the electrical double layer, and the BPE shapes the electric field based on its permittivity (lensing effect). 

Figure 1 reveals electric field maxima at each BPE tip and a minimum over the center of each BPE. A 2D surface plot of the electric field strength in the absence and presence of insulating pillars is depicted in supplementary materials Figure S1. Based on the results at a distance of 9 μm above the electrode tips, assuming a Claussius-Mosotti factor of 1, we estimate that the maximum DEP force experienced by a cell (18 μm diameter) near the BPE tips is 15.6, 20.3, and 24.0 pN at the 100, 160, and 275 μm-long BPEs, respectively. These forces fall within the range (1–100 pN) commonly employed for cellular DEP. This result is important because it supports the general conclusion that the DEP force experienced by cells is positively correlated to BPE length.

### 3.2. Correlation of Cell Cluster Size to BPE Length

**Figure 2:**
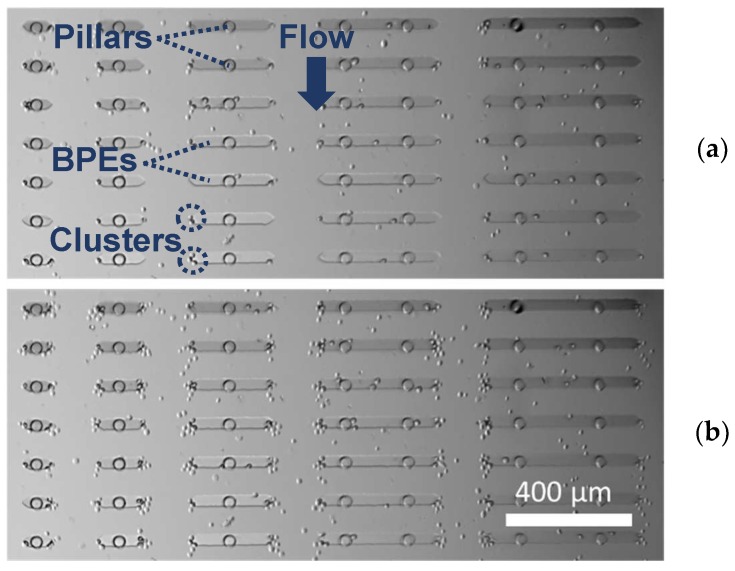
Brightfield micrographs of cells captured at the wireless BPE array. $\Delta U_{tot}$ = 26 V_pp_ (70 kHz). Flow rate, 200 nL/min. Images were obtained (**a**) 1 min and (**b**) 5 min after initiation of $\Delta U_{tot}$.

Figure 2a,b is brightfield micrographs that show a part of the BPE array with cells trapped by pDEP at the BPE tips. This data was obtained as follows. First, DEP buffer in the inlet reservoir was replaced with 20 μL of MDA-MB-231 cells in DEP buffer (2 × 10^6^ cells/mL). Second, DEP buffer was withdrawn from the outlet at a rate of 200 nL/min. Once the cell solution filled the chamber, an AC voltage of 26 V_pp_ (70 kHz) was applied at the driving electrodes flanking the array. Finally, brightfield micrographs of the cells on the BPE array were obtained at *t* = 1 and 5 min, after which the capture voltage was turned off. Once voltage was removed, fluid flow was able to wash the cells away from the electrodes with minimal residual cell adhesion (supplementary materials Movie S1). The micrograph shows a qualitative trend of increased cell cluster size with increasing electrode dimensions, confirming that DEP force is positively correlated to BPE length in practical application. This result is important because it demonstrates that BPE length can be leveraged to control cell cluster size, and using an array with varying BPE length, a range of cluster sizes can be patterned on a single chip.

### 3.3. Cell Cluster Size Controlled by Applied External Voltage at an Array of BPEs of Distinct Lengths

**Figure 3:**
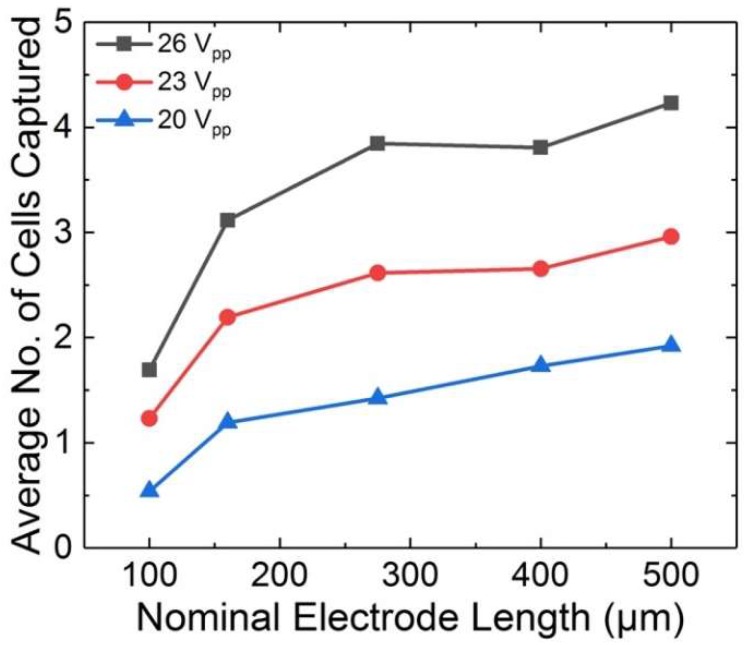
Plot of average cell cluster size at each BPE length (14 total BPEs of each length over two replicates) at a constant flow rate of 200 nL/min for three distinct voltages: $\Delta U_{tot}$ = 20, 23, and 26 V_pp_ (70 kHz). Voltages were chosen to yield cluster sizes of 6 cells or less. Data was obtained at *t* = 5 min.

Figure 3 shows a plot of average cell cluster size at each BPE length for three distinct values of $\Delta U_{tot}$. The experimental procedure was the same as that described in the previous subsection (for the results shown in Figure 2), at a constant flow rate of 200 nL/min and at 70 kHz, with the exception that DEP capture was undertaken at three distinct values of $\Delta U_{tot}$ (20, 23, and 26 V_pp_). The electrode array was imaged 5 min after the application of $\Delta U_{tot}$. Then, the voltage was turned off, and the captured cells allowed to be carried by fluid flow off of the array prior to application of the next voltage. Cell cluster size is reported in Figure 3 as the average number of cells counted at each of 7 BPEs of one length (for each of two capture experiments). The BPEs furthest downstream were excluded because cells were ‘filtered out’ by the upstream electrodes, effectively decreasing the cell concentration. The results of Figure 3 indicate that increased $\Delta U_{tot}$ leads to increased cell cluster sizes at every BPE length. 

For example, at the 160 μm-long BPE, average cluster size was 1.2, 2.2, and 3.1 cells at 20, 23, and 26 V_pp_, respectively. Similarly, clusters contained on average 1.4, 2.6, and 3.8 cells at the 275 μm BPE over the same set of voltages. Therefore, at these electrodes, there was a 2.5-fold and 2.7-fold increase in cluster size with a 30% increase in $\Delta U_{tot}$. At each individual magnitude of $\Delta U_{tot}$, there was an overall upward trend in cell cluster size over a range in BPE lengths from 100 to 500 μm. For example, at 20 V_pp_, the average cluster size increased 3-fold with a 4-fold increase in BPE length (100 to 400 μm). Gains in cluster size with BPE length were likewise modest under the other voltages and lengths investigated. An important conclusion that can be drawn from these results is that cell capture is much more sensitive to $\Delta U_{tot}$ than to BPE length. The influence of the BPE size is more pronounced at the shortest lengths, which we attribute to a uniformly high probability of cell capture at the longer electrodes—these electrodes capture most cells that approach to within a few cell diameters of their tips. At the shortest BPE, the probability is sufficiently low that a significant fraction of cells flowing past are not captured.

It is important to note that error bars indicating the standard deviation in cluster size were excluded from Figure 3 for clarity. The same plot including error bars is available in the supplementary materials (Figure S2). The large standard deviations obtained are attributed to both the stochasticity of the capture process and the fact that the experimental procedure favors cell capture at upstream BPEs. This result indicates that an alternative mode of device operation, such as capture from a quiescent solution at a high cell concentration followed by fluid flow to wash away weakly bound cells, is likely to yield more uniform cluster sizes. The results of a t-test confirmed statistically different cluster sizes between the 100, 160, and 275 μm BPEs for the data of Figure 3 (see supplementary materials).

### 3.4. Cell Cluster Size Controlled by Fluid Flow Rate at Distinct BPE Lengths

**Figure 4:**
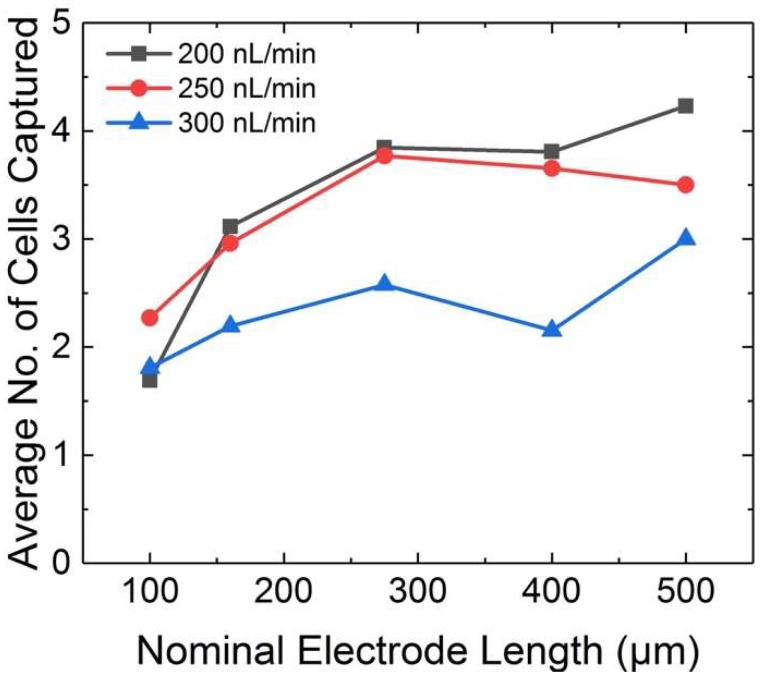
Plot of average cell cluster size at each BPE length (over two replicates) at $\Delta U_{tot}$ = 26 V_pp_ (70 kHz) for three distinct flow rates: 200, 250, and 300 nL/min. Data was obtained at *t* = 5 min.

Figure 4 is a plot of average cell cluster size at each nominal BPE length under constant $\Delta U_{tot}$ of 26 V_pp_ and 70 kHz at three distinct fluid flow rates. The flow rates investigated were 200, 250, and 300 nL/min, which correspond to average linear velocities of 18.5, 23.1, and 27.8 μm/s. The experiment proceeded as described in the previous subsections with the exception that each flow rate was allowed to stabilize for 1 min prior to the application of $\Delta U_{tot}$. Then, the array was imaged after 5 min of cell capture. Finally, the voltage was turned off, and the captured cells allowed to be carried by fluid flow off of the array prior to establishing the next flow rate. The results of Figure 4 show that increased flow rate led to smaller average cell cluster sizes at every BPE length. For example, at several electrode lengths investigated, a 1.5-fold increase in flow rate lead to a 30%–50% reduction in cluster size. Based on these results, we conclude that fluid flow has a more pronounced effect on cell capture than BPE length. Therefore, an array of BPEs can be employed to define cluster sizes that are narrowly distributed within a range set by the voltage and flow rate.

### 3.5. Time Dependence of Cell Cluster Size

**Figure 5:**
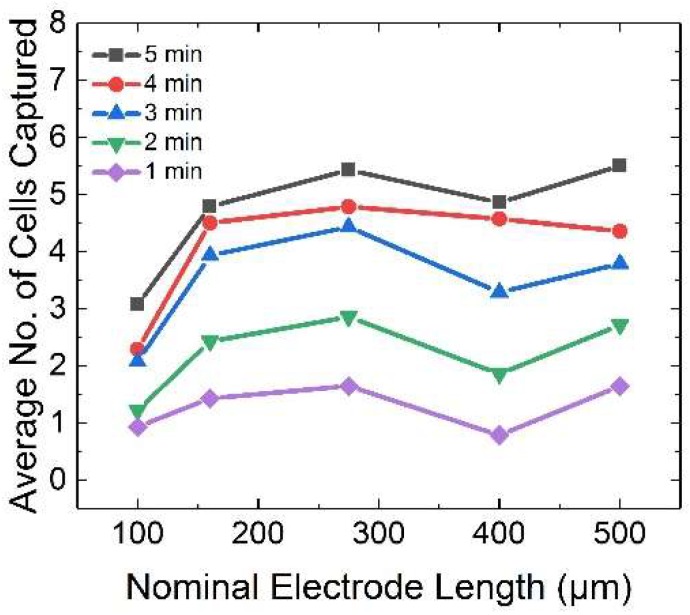
Plot of average cell cluster size at each BPE length at $\Delta U_{tot}$ = 26 V_pp_ (70 kHz) and 200 nL/min obtained at 1 min intervals, up to *t* = 5 min, after initiation of driving voltage.

Next, the time dependence of cell capture was investigated. Figure 5 is a plot of average cell cluster size at each BPE length at 1 min time intervals over a period of 5 min (where *t* = 0 is the initiation of driving voltage, $\Delta U_{tot}$ = 26 V_pp_ (70 kHz)). The flow rate was held constant at 200 nL/min. The results of Figure 5 show that cluster size at each electrode length increases over time. The rate of accumulation appears to taper off at later intervals, which is in agreement with well-established findings that indicate pearl chain length increases with $t^{1/2}$ [43]. In the presence of fluid flow, a balance between *F*_DEP_ and *F*_drag_ defines a steady state cluster size beyond which additional cells are too weakly bound to be retained. This relationship implies that longer capture times favor more uniform cluster sizes (supplementary materials Figure S3).

### 3.6. Control over Cluster Size at Higher Voltage, Flow Rate, and Duration

Finally, based on the results presented in the previous subsections, we hypothesized that a greater dependence of cluster size on BPE length could be achieved at a higher flow rate and voltage. For example, the results of Figure 3 showed a steeper decrease in cluster size at shorter BPEs, which we attributed to the probability of capture at the longer BPEs being too uniformly high to be distinguishable at the flow rate employed. In other words, *F*_drag_ presented an insufficient challenge to *F*_DEP_ at the longest electrodes. Second, we attributed part of the large standard deviation to a ‘filtration effect’ in which upstream electrodes depleted cells from the solution, thereby preventing their capture at downstream electrodes. Based on these two results, we anticipated that a higher flow rate could accomplish more uniform cluster size with a clearer dependence on BPE length over the entire range of lengths investigated. We further hypothesized that a greater voltage would be required to maintain on average one cell at the shortest BPEs (100 µm). 

**Figure 6:**
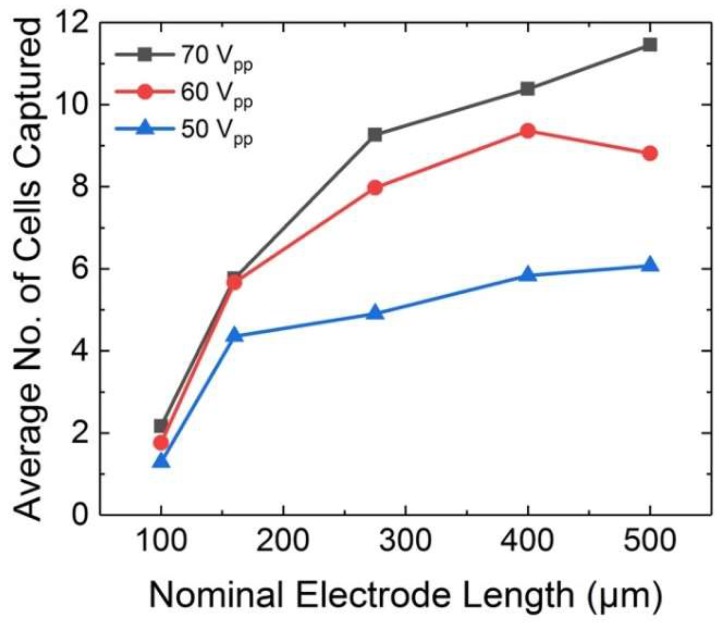
Plot of average cell cluster size at each BPE length (over two replicates) at $\Delta U_{tot}$ = 50, 60, 70 V_pp_ (70 kHz) and 500 nL/min obtained at *t* = 5 min after initiation of driving voltage.

Figure 6 is a plot of average cell cluster size at each BPE length obtained at a flow rate of 500 nL/min at *t* = 5 min after application of $\Delta U_{tot}$ (50, 60, and 70 V_pp_, 70 kHz). As anticipated, these results show a steeper dependence of cell cluster size on BPE length than obtained at lower applied voltage and flow rate. For comparison, in the data obtained at 26 V_pp_ and 200 nL/min (Figure 3), cluster size ranged from 3 to 5.4 cells (a 1.8-fold difference), while here, the average number of cells per cluster extends from 1.3 to 11.5 (nearly 10-fold increase across the array). This result is important because it reveals that the dependence of cluster size on BPE length is most pronounced under conditions that yield sufficient competition between DEP force and flow rate. A t-test revealed that, under the conditions employed in Figure 6, differences in cluster sizes between 100, 160, and 275 μm BPEs were statistically significant (see supplementary materials).

## 4. Conclusions

In conclusion, we have demonstrated that cell cluster size is dependent on BPE length, and varies more steeply where *F*_drag_ presents a challenge to *F*_drag_ that distributes the probability of cell capture at each BPE length in the array. This BPE-length effect is less pronounced than that achieved by altering the applied voltage ($\Delta U_{tot}$) or the fluid flow rate. Therefore, an array of BPEs can be utilized to pattern cells such that the range of cluster sizes is defined by voltage and flow rate, while BPE length defines distinct cluster sizes within that range. We have also shown that the rate of accumulation of cells tapered over time, which indicates that longer capture times reduce time-dependent and BPE-to-BPE variability in the number of cells captured. The reported approach is advantageous in that cell patterning is achieved without using physical constraints or adhesive interactions, which may interfere with cell function, and that DEP-based selectivity is both broad and tunable. Our ongoing research efforts are focused on capturing cells from a hydrogel precursor solution such that subsequent crosslinking of the gel will anchor the cell clusters for on-chip culture, incubation, and analysis. Further, while the current study focused on an operational regime that results in small clusters (<12 cells each), we anticipate that BPE length can be employed as an experimental handle to tune the size of larger clusters as well. Finally, we anticipate that this patterning approach may be amenable to integration with existing methods of electrochemical sensing at BPEs for the characterization of cells.

## Supplementary Materials

The following are available online at https://www.mdpi.com/2072-666X/10/4/271/s1, Figure S1: Surface plot (top view) showing the electric field strength in the (a) absence and (b) presence of pillars (filled white circles) in a plane 9.0 μm above the BPE array under a potential bias leading to a spatially averaged electric field of 4546 V/m. Line plot taken along the midline of the third row of BPEs in the (c) absence and (d) presence of pillars. Figure S2: The data of Figure 2 (main text) plotted with error bars that indicate the standard deviation in the number of cells captured. Plot of the average number of cells captured at each BPE length at $\Delta U_{tot}$ = 20, 23, and 26 V_pp_ (70 kHz). Flow rate, 200 nL/min. Data obtained at *t* = 5 min after initiation of driving voltage. Figure S3: The data of Figure 4 (main text) plotted with error bars that indicate the standard deviation in the number of cells captured. Plot of the average number of cells captured at each BPE length at $\Delta U_{tot}$ = 26 V_pp_ (70 kHz). Flow rate, 200 nL/min. Data obtained at *t* = 1 and 5 min after initiation of driving voltage. Figure S4: The data of Figure 5 (main text) plotted with error bars that indicate the standard deviation in the number of cells captured. Plot of the average number of cells captured at each BPE length at $\Delta U_{tot}$ = 50, 60, 70 V_pp_ (70 kHz). Flow rate, 500 nL/min. Data obtained at 5 min after initiation of driving voltage. Table S1: Table of *p*-values comparing cell cluster sizes at pairs of electrodes lengths as listed at the top of each column. Generated using a one-tailed t-test assuming unequal variances. Movie S1: Video microscopy showing cells held by DEP at the BPE array. The cells are subsequently carried away by fluid flow after the applied voltage is removed.
