# Supplementary material for: Defining Cell Cluster Size by Dielectrophoretic Capture at an Array of Wireless Electrodes of Several Distinct Lengths
Source: Micromachines (Basel). 2019 Apr 23;10(4):271. doi: 10.3390/mi10040271 (PMC6523886; doi:10.3390/mi10040271)
Supplement: Supplementary file 1 [file micromachines-10-00271-s001.zip › supplementary-for publish/Supporting Information/Supplementary Material.docx]

Supplementary Materials: Defining Cell Cluster Size by Dielectrophoretic Capture at an Array of Wireless Electrodes of Several Distinct Lengths

Joseph T. Banovetz ^1^, Min Li ^1^, Darshna Pagariya ^1^, Sungu Kim ^1,2^, Baskar Ganapathysubramanian ^2^ and Robbyn K. Anand ^1,^*

^1^ Department of Chemistry, Iowa State University, 1605 Gilman Hall, 2415 Osborn Drive, Ames, IA 50011, USA; joebano@iastate.edu (J.T.B.); minl@iastate.edu (M.L.); darshna@iastate.edu (D.P.); skim@iastate.edu (S.K.)

^2^ Department of Mechanical Engineering, Iowa State University, 2043 Black Engineering, 2529 Union Drive, Ames, IA 50011, USA; baskarg@iastate.edu

***** Correspondence: rkanand@iastate.edu; Tel.: +1-515-294-8944

**Simulation of the electric field distribution over an array of BPEs.** Figure S1 shows the results of finite element simulations of the electric field strength in the solution at a distance of 9.0 μm above a BPE array in the absence (Figure S1a,c) and presence (Figure S1b,d) of insulating pillars. These pillars represent the PDMS pillars that support the roof of the microchamber employed in the DEP cell capture experiments. Two graphical representations are shown – a surface plot taken from a top view (Figure S1a,b) and a line plot (Figure S1c,d) taken along the midline of the middle row of BPEs. This simulation follows the same parameters outlined in the main text for the generation of the results presented in Figure 1. The data of Figure 1 (main text) is shown here again in Figure S1c for ease of comparison to Figure S1d (with pillars).

| 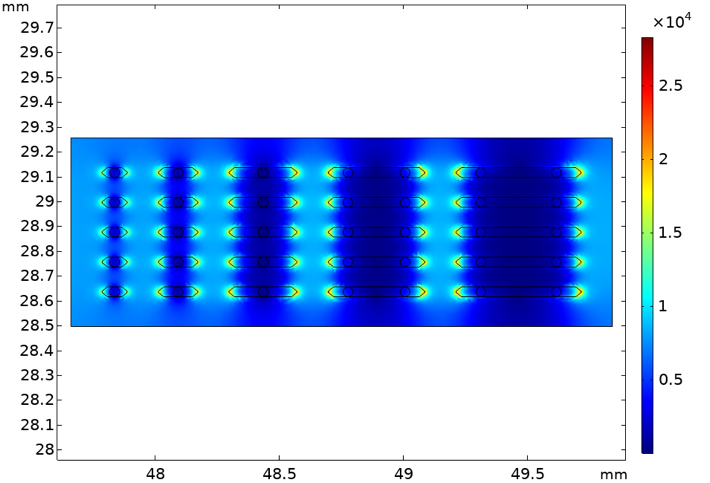 | 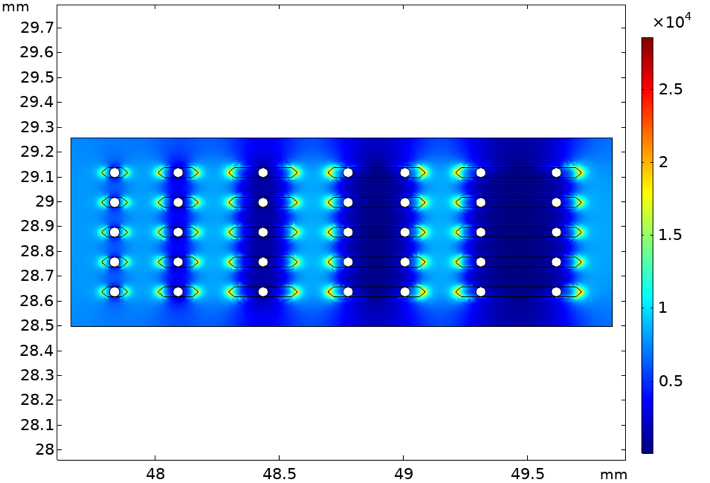 |
| --- | --- |
| (**a**) | (**b**) |
| 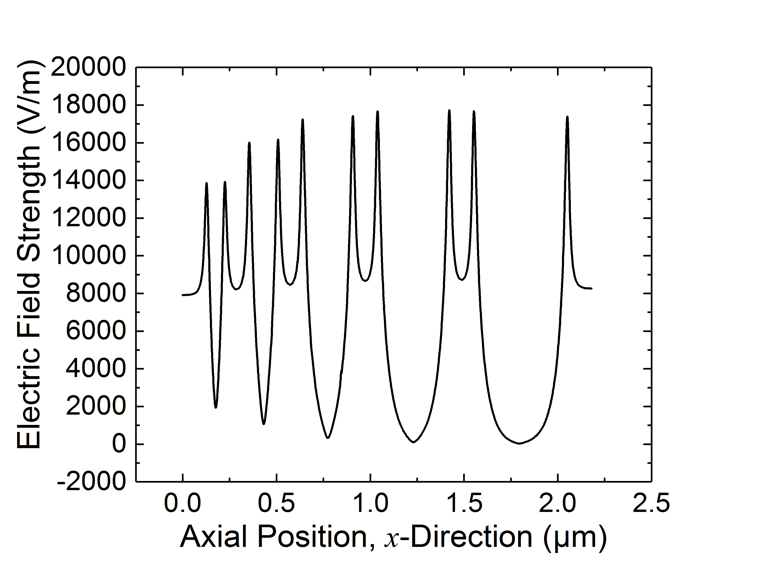 | 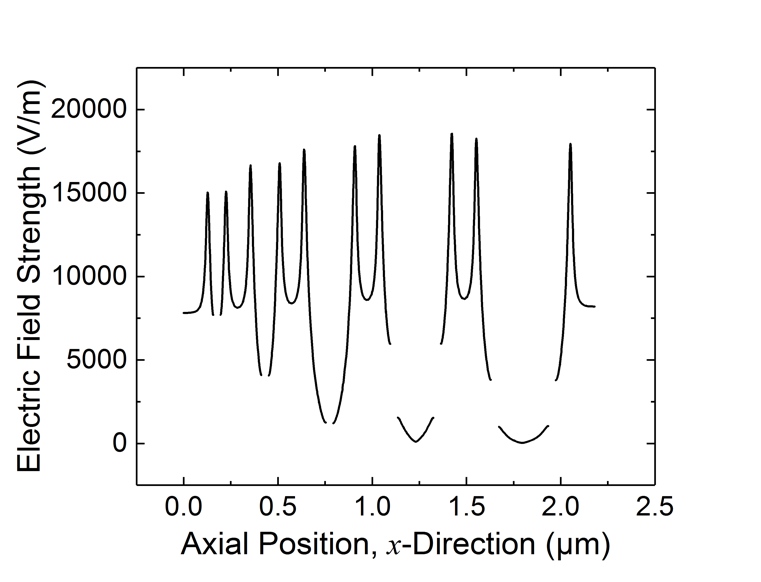 |
| (**c**) | (**d**) |

**Figure S1.** Surface plot (top view) showing the electric field strength in the (**a**) absence and (**b**) presence of pillars (filled white circles) in a plane 9.0 μm above the BPE array under a potential bias leading to a spatially averaged electric field of 4546 V/m. Line plot taken along the midline of the third row of BPEs in the (**c**) absence and (**d**) presence of pillars.

**Cell cluster size as a function of BPE length and driving voltage.** Figure S2 is a plot of the average number of cells captured as a function of BPE length for three distinct magnitudes of driving voltage ($\Delta U_{tot}$ = 20, 23, and 26 V_pp_ (70 kHz)). The flow rate was 200 nL/min and the data was obtained at *t* = 5 min after initiation of the driving voltage. The data is the same as that presented in Figure 2 (main text) but is plotted here with error bars representing one standard deviation in the number of cells captured.


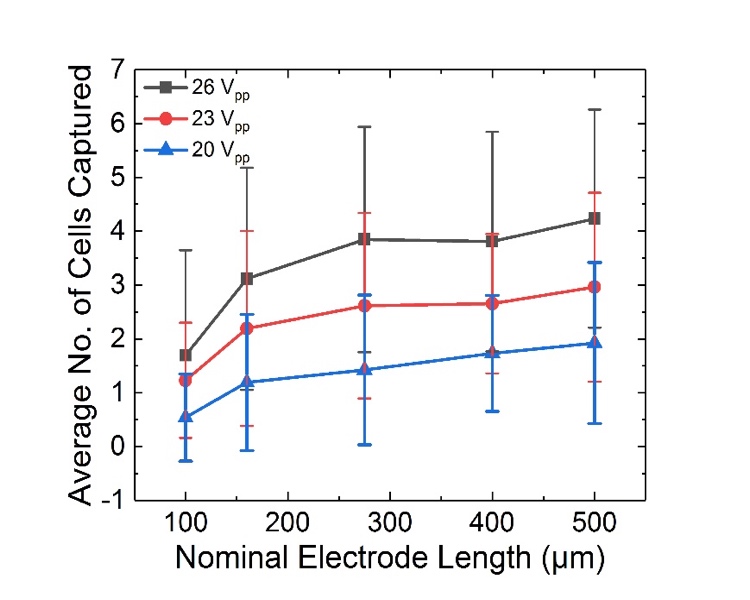


**Figure S2.** The data of Figure 2 (main text) plotted with error bars that indicate the standard deviation in the number of cells captured. Plot of the average number of cells captured at each BPE length at $\Delta U_{tot}$ = 20, 23, and 26 V_pp_ (70 kHz). Flow rate, 200 nL/min. Data obtained at *t* = 5 min after initiation of driving voltage.

**Standard deviation of cell cluster size as a function of BPE length and capture time.** Figure S3 is a plot of the average number of cells captured as a function of BPE length at 26 V_pp_ (70 kHz) and 200 nL/min, and obtained at *t* = 1 and 5 min after initiating the driving voltage. The data is the same as the corresponding time points presented in Figure 4 (main text), here with error bars (one standard deviation). At *t* = 1 min, the standard deviations are 115, 98, 61, 102, and 91% of the average cluster size, from shortest to longest electrode. These values decrease to 62, 32, 25, 32 and 32%, respectively, at *t* = 5 min. An important conclusion that can be drawn from this data is that the percent standard deviation in the cell cluster size decreases with time at all BPE lengths.


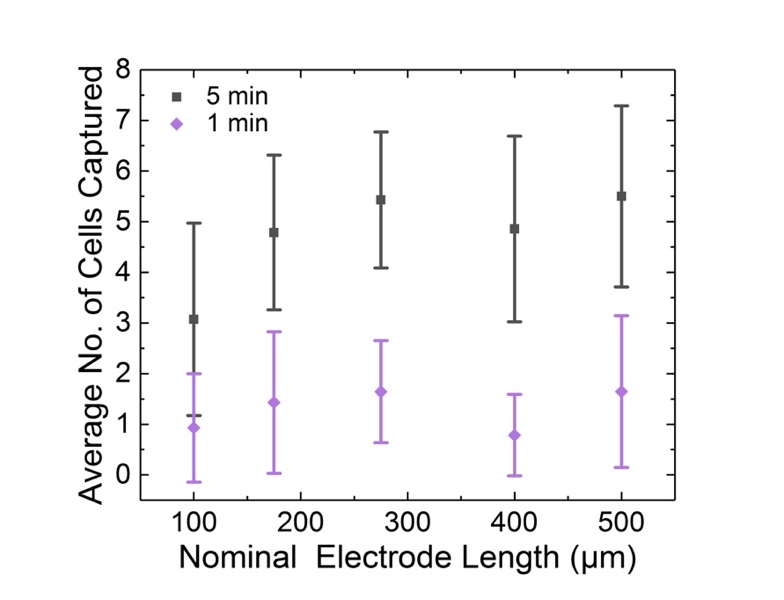


**Figure S3.** The data of Figure 4 (main text) plotted with error bars that indicate the standard deviation in the number of cells captured. Plot of the average number of cells captured at each BPE length at $\Delta U_{tot}$ = 26 V_pp_ (70 kHz). Flow rate, 200 nL/min. Data obtained at *t* = 1 and 5 min after initiation of driving voltage.

**Standard deviation of cell cluster size as a function of BPE length and voltage.** Figure S4 is a plot of the average number of cells captured as a function of BPE length at 50, 60, and 70 V_pp_ (70 kHz) and 500 nL/min, and obtained at *t* = 5 min after initiating the driving voltage. The data is the same as that presented in Figure 5 (main text), here with error bars (one standard deviation).


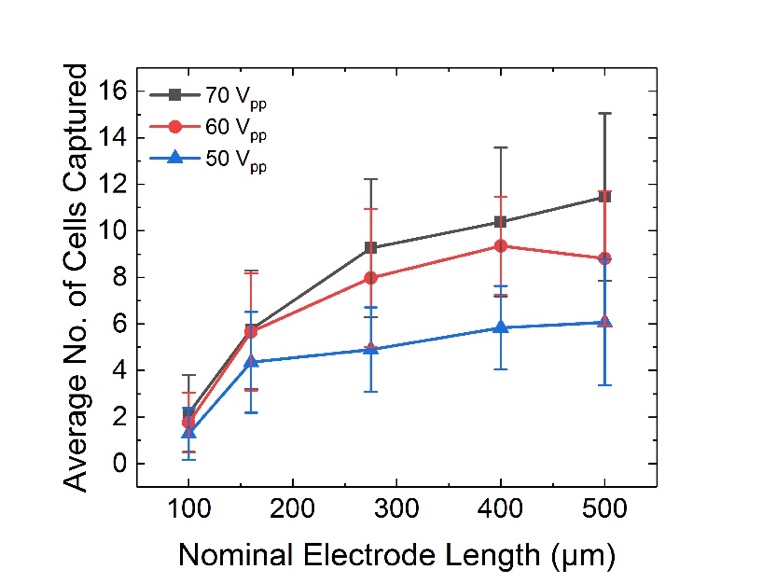


**Figure S4.** The data of Figure 5 (main text) plotted with error bars that indicate the standard deviation in the number of cells captured. Plot of the average number of cells captured at each BPE length at $\Delta U_{tot}$ = 50, 60, 70 V_pp_ (70 kHz). Flow rate, 500 nL/min. Data obtained at 5 min after initiation of driving voltage.

**Statistical evaluation of the differences in cluster sizes at BPEs of distinct lengths.** Table S1 shows the *p*-values obtained for a paired *t*-test for pairs of BPE lengths for the experimental data shown in Figures 2 and 5 (main text). Note that at all voltages employed, the difference in cluster size between the 100 μm and 400 μm BPE (last column, Table S1) was statistically significant.

**Table S1.** Table of *p*-values comparing cell cluster sizes at pairs of electrodes lengths as listed at the top of each column. Generated using a one-tailed t-test assuming unequal variances.

| **Driving Voltage (V_pp_)** | **100–160 µm** | **160–275 µm** | **275–400 µm** | **400–500 µm** | **100–400 µm** |
| --- | --- | --- | --- | --- | --- |
| 20 | 0.023 | 0.14 | 0.27 | 0.42 | 1.5E-4 |
| 23 | 0.018 | 0.16 | 0.47 | 0.33 | 1.3E-4 |
| 26 | 4.0E-03 | 0.11 | 0.50 | 0.25 | 6.3E-6 |
| 50 | 1.0E-11 | 0.11 | 0.010 | 0.32 | 3.8E-15 |
| 60 | 5.0E-13 | 1.1E-04 | 7.9E-03 | 0.16 | 4.8E-21 |
| 70 | 3.6E-11 | 0.06 | 0.050 | 0.08 | 3.2E-22 |

Statistical evaluation of data obtained at higher voltages (50, 60, and 70 V_pp_). A *t*-test was performed on each adjacent data point (Figure 5, main text) to determine statistical significance. At every voltage, the *p*-value (Table S1) for the comparison between the 100 and 160 μm was less than 1 × 10^−10^. Between the 160 and 275 μm electrodes, the *p*-values ranged from 1.0 × 10^−4^ to 0.10, between 275 and 400 μm from 7 × 10^−3^ to 0.49, and between 400 and 500 μm from 0.071 to 0.31. Overall, the statistical analysis shows clear differences between cell cluster sizes at the three shortest electrodes and varying results between 275, 400, and 500 μm electrodes. These results contrast those obtained at the lower voltages and flow rates, which exhibited less statistically clear differences.

Statistical evaluation of data obtained at lower voltages (20, 23, and 26 V_pp_*).* A t-test was performed on each adjacent data point to determine statistical significance. The *p*-values for the differences between the 100 μm and 160 μm electrodes varied from 0.004 to 0.15, and between 160 μm and 275 μm from 0.06 to 0.16. For the other electrodes, the *p*-values were significantly higher, indicating no statistical differences in cell cluster size between the 275, 400, and 500 μm electrodes. This analysis confirms the trend observed in Figure 2 (main text), in which cluster size increases between the 100, 160, and 275 μm electrodes and stays relatively constant at the 400 and 500 μm electrodes.

**Demonstration of cells removal from the BPE array by fluid flow following cell capture.** A video (Movie S1) showing the removal of captured cells by fluid flow after the applied voltage is turned off is included in the electronic Supporting Information. The experiment proceeded as follows. First, MDA-MB-231 cells were subjected to pDEP at the BPE array under an applied voltage of 26 V_pp_ and 70 kHz at a flow rate of 200 nL/min. After 5 min of cell capture, the flow was stopped and the voltage was simultaneously removed. This condition was maintained for 30 s, after which video capture began. After an additional 30 s, fluid flow was resumed at 200 nL/min and the cells were carried by the fluid off of the BPE array. This result confirms that cells do not adhere (stick) irreversibly to the electrodes and glass substrate.
